# Mapping and Modelling Malaria Risk Areas Using Climate, Socio-Demographic and Clinical Variables in Chimoio, Mozambique

**DOI:** 10.3390/ijerph15040795

**Published:** 2018-04-19

**Authors:** Joao L. Ferrao, Sergio Niquisse, Jorge M. Mendes, Marco Painho

**Affiliations:** 1 Faculdade de Engenharia, Universidade Católica de Moçambique, Chimoio 20106, Mozambique; 2 GIS Department, Faculdade de Economia e Gestão, Universidade Católica de Moçambique, Beira 2107, Mozambique; sniquisse@ucm.ac.mz; 3 NOVA Information Management School, Universidade Nova de Lisboa, 1000-260 Lisboa, Portugal; jmm@novaims.unl.pt (J.M.M.); painho@novaims.unl.pt (M.P.)

**Keywords:** modelling, mapping, malaria risk, public precision health

## Abstract

*Background*: Malaria continues to be a major public health concern in Africa. Approximately 3.2 billion people worldwide are still at risk of contracting malaria, and 80% of deaths caused by malaria are concentrated in only 15 countries, most of which are in Africa. These high-burden countries have achieved a lower than average reduction of malaria incidence and mortality, and Mozambique is among these countries. Malaria eradication is therefore one of Mozambique’s main priorities. Few studies on malaria have been carried out in Chimoio, and there is no malaria map risk of the area. This map is important to identify areas at risk for application of Public Precision Health approaches. By using GIS-based spatial modelling techniques, the research goal of this article was to map and model malaria risk areas using climate, socio-demographic and clinical variables in Chimoio, Mozambique. *Methods*: A 30 m × 30 m Landsat image, ArcGIS 10.2 and BioclimData were used. A conceptual model for spatial problems was used to create the final risk map. The risks factors used were: the mean temperature, precipitation, altitude, slope, distance to water bodies, distance to roads, NDVI, land use and land cover, malaria prevalence and population density. Layers were created in a raster dataset. For class value comparisons between layers, numeric values were assigned to classes within each map layer, giving them the same importance. The input dataset were ranked, with different weights according to their suitability. The reclassified outputs of the data were combined. *Results*: Chimoio presented 96% moderate risk and 4% high-risk areas. The map showed that the central and south-west “Residential areas”, namely, Centro Hipico, Trangapsso, Bairro 5 and 1° de Maio, had a high risk of malaria, while the rest of the residential areas had a moderate risk. *Conclusions*: The entire Chimoio population is at risk of contracting malaria, and the precise estimation of malaria risk, therefore, has important precision public health implications and for the planning of effective control measures, such as the proper time and place to spray to combat vectors, distribution of bed nets and other control measures.

## 1. Background

Malaria is an ancient disease and a major public health concern in Africa. Approximately 3.2 billion people remain at risk of contracting malaria, and 214 million new cases of malaria were reported in 2015, resulting in 428,000 deaths. Most cases (88%) occurred in the WHO African Region, followed by the South-East Region (10%) and WHO Eastern Mediterranean Region (2%). Approximately 80% of cases occurred in just 15 countries, mainly located in Africa. Combined, these high-burden countries recorded a slower than average reduction in malaria incidence and mortality [1], and Mozambique was among them.

Mozambique was recently ranked fifth in Africa for the number of malaria cases [2]. The disease is a major cause of morbidity and mortality, especially among children [3], and the entire population is at risk of contracting the disease since it is endemic with seasonal peaks during and after the rainy season, which is between November and March [4,5].

Eradicating malaria is, therefore, one of Mozambique’s main priorities and is recognized as critical to achieve the 2030 Agenda for Sustainable Development [6]. The environment and climate conditions highly influence malaria transmission, although their effects are often not linear. The malaria-climate relationship varies among areas covered by different agro-ecological zones [6]; thus, resources for control have to be spread over time and space. An estimated 80 to 90% of malaria cases are related to environmental factors [7,8,9]. The level of prevalence can be predicted based on the established relationship between malaria prevalence and environmental data.

Temperature affects the development of malaria as the parasite does not develop below 18 °C and over 40 °C [10,11]. The highest proportion of vectors surviving the incubation period is observed at temperatures between 28 °C and 32 °C [12]. Precipitation is another key player in malaria occurrence; increased precipitation can provide more breeding sites for mosquitoes, but excess rain can also destroy breeding sites [13,14]. Altitude can indirectly influence the distribution and spread of malaria via its effect on temperature. For every 200 m increase in altitude, the temperature decreases by 1 °C [15]. Highlands are colder and lowlands are warmer; at certain altitudes, malaria transmission does not occur due to extreme temperatures, which are not favourable to the mosquito or the life cycle of the parasite [10]. For smaller regions, topography remains the single most important aspect that defines large scale differences in malaria risk because climate variables change little over the limited range of latitude [16]. In Mozambique and Malawi, elevation is associated with malaria prevalence; at an elevation of <650 m, the prevalence was higher than that between 650 and 1100 mm [17,18].

Slope combined with precipitation levels at a certain location may influence the dispersion of malaria. Flat areas on the ground are more prone to accumulate water, creating dam rainwater and increasing the risk of malaria. In Ghana, swampy areas and banana production in the proximity of villages are strong predictors of a high malaria incidence [19].

Land cover is another factor in malaria occurrence. In Kenya, the association between land cover type and presence of anopheline larvae was statistically significant; overall, the highest proportions of anopheline-positive habitats occurred in pastures (33%) and farmlands (32%), followed by swamp habitats (23%) [20]. In Ghana, an increase in a forested area of 10% was associated with a 47% decrease in malaria incidence. Different cultivations in the vicinity of homesteads were related to childhood malaria in rural areas [19,20]. Marsh clearance, dam construction and crop cultivation increase the risk of malaria at the local scale. Open, treeless habitats have a lower malaria transmission risk compared with forest sites [16].

The effectiveness of intervention measures against malaria can be determined by the Euclidian distance of a place from roads. In Zambia, for every 500 m increase in distance from the road, there was a corresponding 5% increase in malaria-positive households [21]. In Kenya, roads were found to have the fewest number of anopheline habitats (15%), whereas habitats in forests had an 18% rate [20].

The distribution of water bodies is a major factor that influences malaria occurrence and case distributions. Water bodies play a very important role as larval breeding sites for malaria mosquitoes [22]. Therefore, identification of water body sites is a direct indicator for malaria risk occurrences. The Euclidian distance to a water body is a determinant of the malaria risk incidence [23]. A study carried out in China indicated that populations living within 60 m of water bodies had a higher risk of contracting malaria [24].

In terms of malaria breeding, the following statement was made in 1934: “it may safely be inferred that the influence of any production from breeding place within 0.81 km radius will be felt there in, at radii of 1.61 km the influence may be doubtful, and ordinarily at radii of more than 1.61 km the influence may be expected to be nil” [25]. Recent studies have indicated that mosquitoes fly no more than 170 m after ingesting a blood meal [26] and that a hungry mosquito will fly up to 1.5 km [27].

In Chimoio, a weak positive correlation was found between malaria cases and population density [28]. The mosquito breeding, feeding, and resting behaviours are often associated with vegetation [18]. 

A number of vegetation indices have been used in remote sensing, but the most used index to enhance the vegetation areas is the Normalized Difference Vegetation Index (NDVI). The measurement of NDVI is from −1 to 1; if a value is close to 0, there is little vegetation in the area. When the value is close to 1, there is more vegetation in the region [16]. In Brazil, most domiciles with more than five notified cases are located near areas with high NDVI values, and variations in the photosynthetic productivity of vegetation are strongly related to variations in malaria transmission [29].

Chimoio is the capital of Manica Province in the Centre of Mozambique. Very little research on malaria has been carried out in Chimoio. Malaria is increasing in the suburbs, and urban areas present fewer malaria cases than rural areas. The annual overall average of malaria incidence is 20.1%, and the Attributable Fraction (AF) of malaria is 16%. Children under five are three times more prone to malaria than adults [28], and 11.7% of the total annual deaths are due to malaria [30].

The two most important climate factors that influence malaria in Chimoio were found to be the relative humidity and minimum temperature, and they showed positive high correlations with climate [8]. In regard to the spatial epidemiology of malaria, recent studies have benefited from the great progress in the development of Geographic Information Systems (GIS). The health practitioner and/or researcher’s ability to locate the precise position of a disease in their area allows for the creation of maps of the spatial variability, and they can incorporate as many variables as can be measured [31].

Precision public health strategies are based on a specific site by observing, measuring and responding to inter and intra-region variability in malaria trends. This makes statistical and computational treatments quite involved and can lead to decision support systems that can help to eradicate malaria, optimize resources and minimize the impact on the environment [32]. The decisions can be in areas such as the right time and place for spraying, correct site to build a water body, correct time and place for drainage and other relevant activities for malaria control and eradication.

Targeting vector control in high-risk areas, focusing on asymptomatic and symptomatic infections and managing importation risk are needed to control and eradicate the disease. High spatial and temporal resolution maps of malaria risk can support all of these activities [33].

Risk maps can be used for Precision Public Health, but the maps available for malaria were produced at national, regional or continental magnitudes, such as MARA [34,35], and have a limited operational use to support local programme activities.

Malaria risk maps of the country, especially of Chimoio, have not been produced, and they are urgently needed to identify areas at risk by the Public Precision Health approach. By using spatial modelling techniques with GIS, the research goal of this study was to map and model malaria risk areas using socio-demographic, climate, and clinical variables in Chimoio, Mozambique.

## 2. Materials and Methods

### 2.1. Study Area

Chimoio is a municipality located in Manica Province in the central region of Mozambique. The population of Chimoio is presently estimated to be 324,816 [36]. The area is 174 km² and is at an altitude that varies between 513 and 786 m. The Chimoio climate has a warm temperature with dry winters from April to July, hot and dry summers from August to October and hot and humid summers from November to April. The annual average temperature is 21.5 °C, and the annual precipitation is 1143 mm [37]. The major economic activities are agricultural production, livestock growing, general trading, metallurgical industry, food industry, tourism, telecommunication, banking and insurance and energy supply [38]. 

### 2.2. Materials

(i) Bioclimatic (1950 to 2000) (WorldClim) [39].
(ii) ArcGIS 10.2. [40].
(iii) 30 m × 30 m Landsat image 8 [41]. Table A1 (Appendix A) presents the spectral characteristics of Landsat 8, accessed on 18 April, 2016.
(iv) 30 m × 30 m digital elevation model [42].

### 2.3. Methods

**Figure 1:**
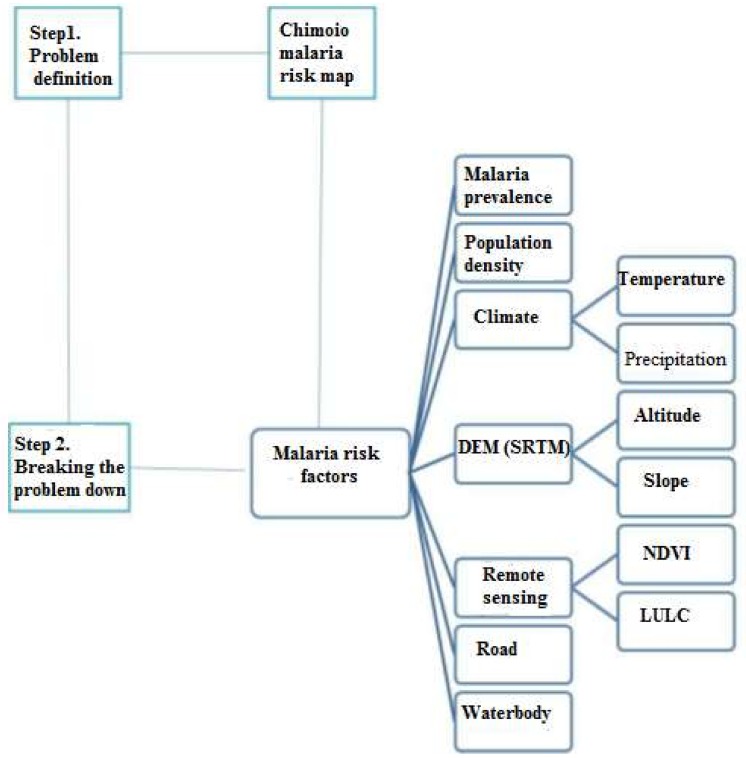
Schematic representation of the data flow and analysis for generating a malaria risk map for Chimoio. DEM/SRTM = Digital elevation model/Shuttle Radar Topography Mission, NDVI = Normalized digital difference index, LULC = Land use and land cover.

Figure 1 presents the schematic representation of the data flow and analysis for generating a malaria risk map for Chimoio.

A conceptual model to solve spatial problems was used to create the Chimoio Map risk [43]. The process involved the following steps:



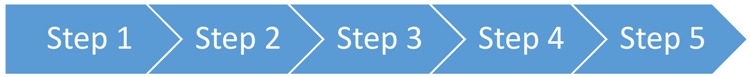

State the ProblemBreak down the problemAnalytical hierarchical processAnalysisResult verification

#### 2.3.1. Step 1. In This Stage, the Problem Was Stated As

Mapping the malaria risk of Chimoio.

#### 2.3.2. Step 2. Breaking down the Problem

Table 1 presents the malaria factors; their weight and classification; and the rationale for the classification adapted from the literature from Zimbabwe, Tanzania and Latin America [16,44,45,46]. For this stage, the input data set or malaria risk factors were as follows: average temperature (Tmean), precipitation (PP), altitude (Alt), slope (SLP), distance to water body (DTWB), distance to road (DTR), Normalized Difference Index (NDVI), land use and land cover (LULC), malaria prevalence (Mal prev) and population density (pop dens).

##### Average Temperature (Tmean)

Long-term minimum and maximum temperatures were extracted from the Bioclim (WorldClim), 1970 to 2000 [40], and the average temperature was calculated. In this study, average temperatures below 22 °C were classified as low risk for malaria transmission, those from 22 °C–32 °C were classified as high-risk for malaria transmission, and those above 32 °C were classified as of moderate risk. 

##### Precipitation (Prec)

Precipitation data were extracted from the Bioclim Data [40]. In the study, areas that received precipitation less than 450 mm were classified as low risk, those that received precipitation between 450 to 700 mm were classified as a moderate risk, and those that received precipitation over 700 mm were classified as having high risk.

##### Altitude (Alt)

A digital elevation model at a 30 m × 30 m resolution was used to estimate the altitude [43]. Areas below 200 m (lowlands) were classified as being at the highest risk for malaria occurrence, areas between 201 to 500 m (uplands) were classified as having moderate risk and areas over 500 m (midlands) were classified as having the lowest risk of malaria exposure. 

##### Slope (SLP)

The slope was derived from the 30 m × 30 m digital elevation model [43], which was obtained from the spatial analysis tool from ArcGIS. In the study, areas from 0 to 5 degrees were classified as being high-risk, those from 5 to 15 degrees were classified as of moderate risk, and those over 15 degrees were classified as having the lowest risk.

##### Land Cover and Land Use (LULC) 

Land use and land cover data were retrieved from the most recent (April 2016) 30 m × 30 m Landsat satellite image [42]. The image was reclassified into different LULC classes using the manual training sampling technique and maximum likelihood algorithm. Areas with crops, grass and water bodies were classified as having a high risk of malaria. Areas with shrubs and mosaic cover vegetation were classified has having a moderate risk of malaria, while areas with forest, bare, and urban settlements were classified as having the lowest risk of malaria [16]. 

##### Distance from Roads (DTR)

The Euclidean distance to the nearest road was calculated using ArcGIS by classifying a 2016, 30 m × 30 m Landsat image [42]. The distances of sites from the road were then calculated using the measuring distance function in ArcGIS 10.2.2 software. In the study, sites over 5 km from the roads were classified to be at highest risk of malaria, those between 2.6 km and 5 km from roads were classified to be at moderate risk and those less than 2.5 km from the roads were classified as having the lowest risk of malaria infection.

##### Distance to Water Bodies (DTWB)

Distance to the nearest water body was calculated with ArcGIS by classifying a 2016 30 m × 30 m Landsat image for water and undefined zones [42]. The distance from water bodies was then calculated using the measurement distance function in ArcGIS software. In this study, areas with less than 500 m from a water source were classified as high-risk areas of malaria, those between 501 to 1500 m were classified as moderate risk areas, while those above 1500 m from water bodies were classified as low risk areas.

##### Population Density (Pop Dens)

Data on the population density were calculated from the national census population projections for 2014 [37]. Data on the population density were related to administrative units. In the study, sites with over 9000 people/km^2^ were classified to be at the highest risk of malaria, those between 6001 to 9000 people/km^2^ were classified to be at moderate risk, and those with less than 6000 person/km^2^ were classified to be at low risk. 

##### Malaria Prevalence (Mal Prev)

Malaria cases diagnosed by health personnel as described elsewhere [28] were used. In the study, over 21% were classified as being the highest malaria risk areas, between 14 and 21% were classified as being moderate malaria risk areas and less than 14% were classified as being the lowest risk areas. Data on malaria prevalence were related to administrative units. 

##### Normalized Difference Vegetation Index (NDVI)

Vegetation vigour is indicated by NDVI, and higher amounts of green vegetation on the ground indicate higher NDVI. Non-vegetation classes are generally lower than vegetation classes in NDVI. NDVI was extracted from a Landsat image [41]. The NDVI map was grouped into three principal categories: −0.288 to 0, classified as low risk; 0 to 0.25, classified as moderate risk; and 0.255 to 0.986, classified as high risk [16]. The NDVI is presented in Equation (1):

NDVI = (NIR − RED)/(NIR + R)(1)

where NIR is the reflectance in the near infrared band, and R is the reflectance in the red band of satellite data [47].

#### 2.3.3. Step 3: Analytical Hierarchical Process (AHP)

The analytical hierarchical process uses hierarchical structures to represent a problem and makes judgments based on expert panels to derive priority scales [16,48]. In this step, the input datasets were explored to understand their content, and attributes within and between data sets were more important for solving the stated problem and searching for trends in the dataset. To obtain the weights for each individual factor for the map, the following steps were taken:

(a) Formulation of a pair-wise comparison matrix for each of the input variables. The fundamental scale to help in the weighting process was used to develop the pair-wise comparison matrix (Table 1). For the designation of the importance of each variable, they were weighed using a pairwise comparison method, which is one of the components of AHP. Saaty’s pairwise comparison table was used to assist in the weighting process [49].
(b) Establishment of the relative weights of each input variable. In the modelling of the final malaria risk areas, the risk factors do not have the same role and weight. Therefore, to designate the importance of each variable, they were weighted using a pair-wise comparison method from the AHP template worksheet [50].
(c) Checking for consistency in the pairing process [16]. After computing the pair-wise matrix and to measure whether the derived matrix was derived at an acceptable level, a consistency test was calculated using Equation (2):
  (2) $$CI=\frac{\lambda -n}{n-1}$$
  where *n* is the dimension of comparison matrix and l max is the maximum eigenvalue of the comparison matrix. The consistency of a pairwise matrix can be interpreted as shown in Table 2.

For this study, a consistency index of less than 10% was considered sufficient [16]. For a result above 10%, the matrix was revised until reaching an adequate level of acceptance.

#### 2.3.4. Step 4: Performing the Analysis

**Figure 2:**
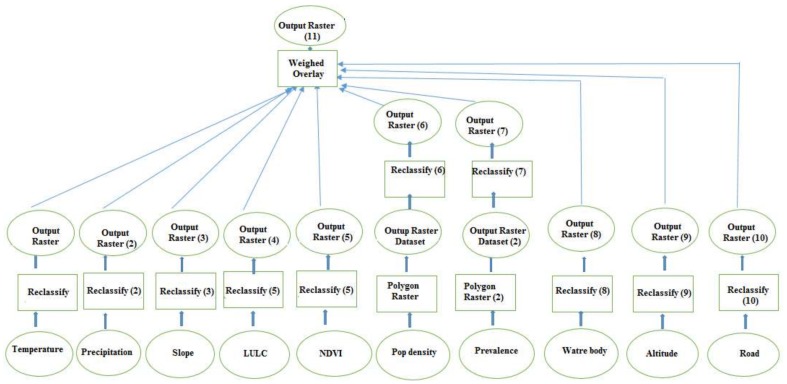
Spatial analysis.

The spatial analysis depicted in Figure 2 was performed. 

Layers for the average temperature (Tmean), precipitation (PP), altitude (Alt), slope (SLP), distance to water body (DTWB), distance to road, NDVI, LULC, malaria prevalence (%) and population density (person/km^2^) were created in a raster dataset. The reclassification was carried out, and all of the measures had the same numeric scale, giving them the same level of importance. 

For the suitability model, reclassified outputs of Tmean, PP, Alt, SLP, DTWB, DTR, NDVI, LULC, Mal prev, and Pop dens were combined. Weights were assigned at the same time by combining the suitability maps. To compare the values of the classes between the layers and numeric values to that of the classes within each map layer, these factors were assigned values from 1 to 3, representing low, moderate and high risk, respectively.

The index overlay method was used, in which each of the input maps was allocated a weight and every class and spatial unit existed in each factor map; therefore, different classes on a single map had different weights and each variable map also had its own weight. The final risk map was produced by summing all of the input variable maps after each had been multiplied by its overall weight, as presented in Equation (3) [16]:

(3) $$S=\frac{\sum \;WiSij}{\sum Wi}$$

where *Wi* is the weight of *i*-th factor map, *Sij* is the *i*-th spatial class weight of *j*-th map, and *S* is the spatial unit value in the output map.

#### 2.3.5. Step 5: Verifying the Result

After obtaining the results of the spatial analysis, the accuracy of the findings was discussed with malaria health personnel form the Provincial Directorate for Health. 

## 3. Results

Table 3 shows the 10 × 10 comparison matrix of the malaria risk factors used in the study. A value of 1 means that the factors under comparison have the same weight and that they equally affect the malaria occurrence. A value of five means the factor in the column is five times more important in malaria risk occurrence than the comparison in the row.

The weights of each factor used for the spatial model to produce the malaria risk map are presented in Table 4. The average temperature (22.4%) and precipitation (20.8%) presented the highest weights, followed by the distance to water body (12.3%) and altitude (10.4%), land use and land cover (8.2%), slope (7.3%), pop dens and malaria prevalence (5.1%), NDVI (4.7%) and distance to road (3.8%). The consistency index for the pair-wise matrix was 9%. 

The special model to produce the malaria risk map formula was:

[(Tmin × 0.224) + (precipitation × 0.208) + (altitude × 0.104) + (slope × 0.073) + LULC × 0.082) + (DTWB × 0.123) + (DTR × 0.038) + (Pop dens × 0.051) + (Mal prev × 0.051) + (NDVI × 0.047)].

**Figure 3:**
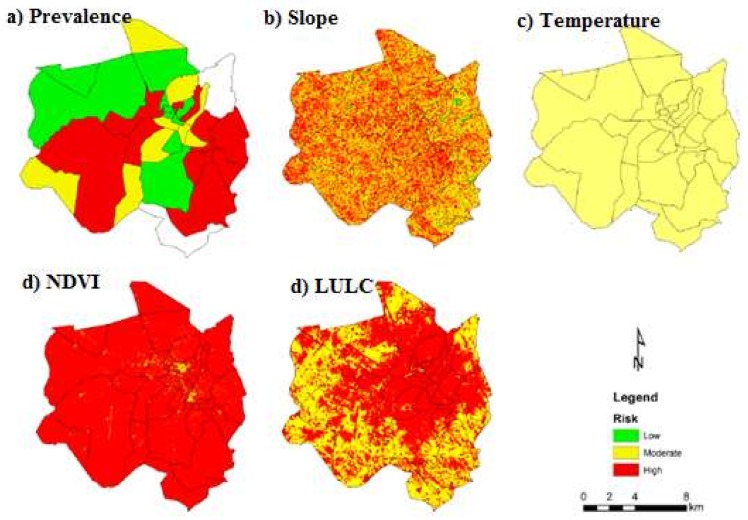
Risk for malaria occurrence. (**a**) Prevalence; (**b**) slope; (**c**) temperature; (**d**) NDVI; (**e**) LULC.

Figure 3 presents the maps of malaria prevalence, slope, temperature, NDVI and LULC. In terms of malaria prevalence, 42% of Chimoio’s areas presented a low risk, 17% a moderate risk and 41% a high risk. For the slope, 2% of the area presented a low risk, 52% a moderate risk and 46% a high risk. For the average temperature, 100% of Chimoio presented a moderate risk. For NDVI, 5% of Chimoio presented a low risk, 12% a moderate risk and 88% a high risk. For LULC, 39% of Chimoio presented a low risk, 4% a moderate risk and 43% a high risk. 

**Figure 4:**
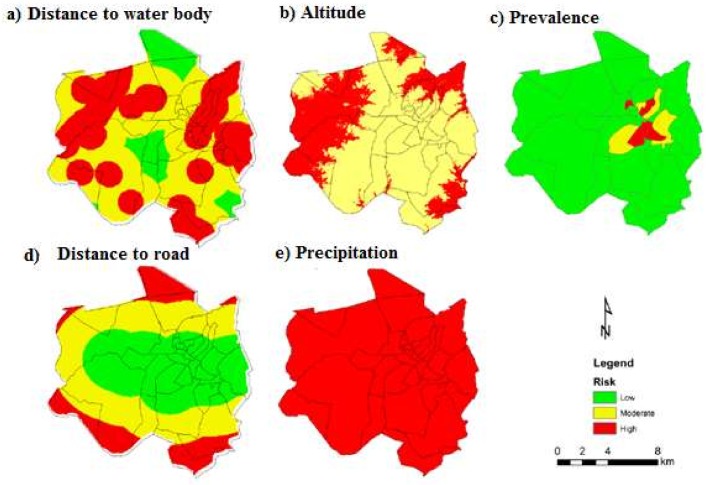
Risk of malaria occurrence. (**a**) DTWB; (**b**) altitude; (**c**) population density; (**d**) distance to road; (**e**) precipitation.

Figure 4 presents the maps of precipitation, altitude, distance to a water body (DTWB), distance to road (DTR), and population density (person/km^2^). For precipitation, Chimoio presented 100% high-risk areas. For altitude, 34% of Chimoio presented a moderate risk and 66% a high risk. For DTWD, 44% of Chimoio presented a low risk, 40% a moderate risk and 16% a high risk. For DTR, 40% of the area presented a low risk, 43% a moderate risk and 17% a high risk. For population density, 92% of Chimoio presented a low risk, 5% a moderate risk and 3% a high-risk areas.

**Figure 5:**
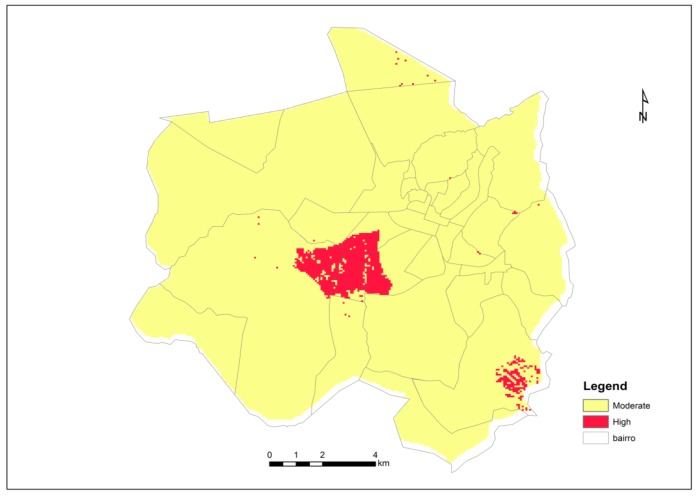
Chimoio risk map for malaria.

Figure 5 presents the Chimoio map risk for malaria after consolidation of the weighted malaria risk factors used in the present study. Chimoio presented 0% of the area with low risk, 96% with moderate risk and 4% with high risk. The map shows that the central and south-west residential areas, namely, Centro Hipico, Trangapsso, Bairro 5 and 1° de Maio, while the rest of the residential areas had a moderate risk of malaria.

## 4. Discussion

Few studies have included ten risk factor variables in geostatistical models for malaria risk mapping. In this study, 100% of Chimoio presented high-risk areas for precipitation, indicating that precipitation is a key player in malaria occurrence and that increased precipitation provides more breeding sites for mosquitoes [9,12,13,14]. For NDVI, 88% of the area presented high risk. Variation of the photosynthetic productivity of vegetation is strongly related to variation in malaria transmission. Similar NDVI results to those found in this study were reported in Brazil [29]. Altitude indicated that 66% of the area was at high risk. At altitudes over 1000 m, malaria transmission decreases due to extreme temperatures, which are not favourable to the mosquito or parasite life cycle [10]. The altitude of Chimoio varies from 513 and 786 m and seems to be favourable for malaria transmission. Similar results were reported in Mozambique [18,28], Swaziland and Malawi [17,22]. 

In this study, 100% of the Chimoio area presented a moderate risk for temperature and 52% for slope. The highest proportion of vectors surviving the incubation period is observed at temperatures between 28 °C and 32 °C [12], and the annual average temperature in Chimoio is 21.5 °C [38]. These observations can explain why the entire Chimoio area is at moderate risk. The slope together with the precipitation levels at a certain location may influence the dispersion of malaria. Flat areas on the ground are more prone to accumulate water, creating rainwater dams, increasing the risk of malaria [18]. The results of this study are similar to studies in Mozambique [51,52] and Ghana [19].

In this study, 92% of Chimoio presented a low risk for population density, 44% for distance to water bodies, and 42% for malaria prevalence and distance to the road. A previous study in Chimoio found a weaker relationship between malaria prevalence and population density [27] compared to studies in China [25]. 

In this study, climatic factors, mean temperature and precipitation presented the highest weights, followed by DTWB (12.3%) and altitude (10.4%), and the other climatic factors presented the least weights. The Mozambique risk map was similar to the findings of this study [34,53,54]. Similar results were also reported in other studies in Zimbabwe, Tanzania, and Latin America [16,46,47,48]. 

The malaria risk map produced by this study differed in many ways from other available models. The area is small (174 km^2^), and the model used ten risk factor variables. The model also used high, sharp and fine spatial and temporal resolutions of risk factors and included climate variable data that impacted factors that affect mosquito proliferation. The model also included human-induced variables, such as the distance from roads, LULC changes, and clinical data. The model is reasonably scaled to present the variance in the malaria risk at the micro-scale level. Few studies have included ten risk factor variables in geostatistical models for malaria risk mapping. Similarly, this approach can also be applied to model and prediction of other environment-driven diseases.

One limitation of the current study is that it does not account for seasonal effects which can impact on disease transmission such as, temporal changes is malaria transmission risk, the environmental conditions that are suited to key stages of the mosquito life cycle, and mosquito numbers that varies throughout the year. Despite the limitations, one great strength of the study is that this is the first specific study in malaria mapping in Chimoio.

## 5. Conclusions

The weights used in this map are consistent with those from several studies, and the map is reliable. The entire population of Chimoio is at a risk of contracting malaria; 96% have a moderate risk and 4% have a high risk. Trees in the Chimoio streets and households are likely resting areas for mosquitoes.

Precise estimation of malaria risk has important precision public health implications and the planning of effective control measures, such as the right time and place to spray to combat vectors, right time to prune trees from homesteads, distribution of bed nets, correct site to build a water body, correct time and place for drainage and other relevant activities for malaria control and eradication.

This study demonstrated the importance of the use of GIS and remote sensing in predicting, mapping and modelling the malaria risk in the Chimoio municipality. More studies should be carried out, such as on bed net usage, the relationship between household presence of trees and malaria and others.

## Figures and Tables

**Table 1:** Classification, weighing and rationale of the malaria risk factors.

Factor	Weight	Class	Influence	Rationale
T mean	0.224	<22 °C	Low	Bellow 22 °C sporogony is not completed
		>28 °C	Moderate	Over 28 °C sporogony is affected
		22–28 °C	High	22–28 °C ideal for incubation
Precipit	0.208	<450 mm	Low	<450 mm is arid, and mosquitoes will not survive
		450–700 mm	Moderate	difficulties in survival >700 mm is wet and
		>1000 mm	Low	inappropriate for mosquito breeding
Altitude	0.123	<200 m	High	<200 m low land and high risk of vector
		200–500 m	Moderate	proliferation, 200 to 500 m upland
		>500 m	Low	>1000 m highlands and low risk of mosquito survival
Slope	0.082	0–5°	High	appropriate conditions for water stagnation
		5–15°	Moderate	
		>15°	Low	>15° inappropriate for water stagnation
LULC	0.082	crop, grass and water bodies	High	Suitable for mosquitoes’ proliferation
		shrubs and mosaic vegetation	Moderate	
		forest, bare, urban	Low	Not suitable for mosquitoes breeding
DTWB	0.123	<500 m	High	The mosquito fly range is 1500 m
		500–1500 m	Moderate	Less than 500 m from WTBD
		>1500 m	Low	the risk of malaria is high
DTR	0.038	<2.5 Km	Lowe	<2.5 km walking distance to clinic
		2.5–5 km	Moderate	2.5 to 5 km clinic can be reached by bicycle
		>5 km	High	<5 km interventions are difficult
Pop dens	0.051	<6000 pers/km^2^	Low	High populated area has higher risk
		6000–9000 pers/m^2^	Moderate	since mosquitoes have abundant
		>9000 pers/km^2^	High	blood meals close by
Malar prev	0.051	<14%	Low	High prevalence areas have higher
		14–21%	Moderate	risk since mosquitoes do not have
		>21%	High	to travel long for a blood meal
NDVI	0.047	−0.2777–0	Low	
		0–0.255	Moderate	
		0.255–1	High	High NDVI is related to high malaria risk

Tmean = average temperature, PP = precipitation, Al t = altitude, SLP = slope, DTWB = distance to water body, DTR = distance to road, NDVI = normalized difference index, LULC = land use and land cover, Mal prev = malaria prevalence, pop dens = population density.

**Table 2:** Nine degree fundamental scale for a pair-wise comparison matrix by Saaty.

Scale	Degree of Preference	Explanation
1	Equal importance	Two factors contribute equally to the objective.
3	Moderate importance of one factor over another	Experience and judgment slightly favour one over the other.
5	Strong or essential importance	Experience and judgment strongly favour one over the other.
7	Very strong importance	Experience and judgment very strongly favour one over the other. It is experience is demonstrated in practice.
9	Extreme importance	The evidence favouring one over the other is of the highest possible validity.
2,4,6,8	Values for inverse comparison	When compromise is needed.

**Table 3:** 10 × 10 Comparison Matrix of the Risk Factors used in the study.

	Tmean	Prec	Alt	Slope	LULC	DTWB	DTR	Pop den	Prev	NDVI
**Tmean**	1.00	1.00	3.00	4.00	4.00	2.00	6.00	4.00	4.00	4.00
**Prec**	1.00	1.00	3.00	4.00	3.00	1.00	7.00	4.00	4.00	4.00
**Alt**	0.33	0.33	1.00	3.00	3.00	1.00	4.00	2.00	2.00	3.00
**Slope**	0.25	0.25	0.33	1.00	1.00	2.00	1.00	3.00	1.00	1.00
**LULC**	0.25	0.33	0.33	1.00	1.00	2.00	2.00	5.00	1.00	1.00
**DTWB**	0.50	1.00	1.00	0.50	0.50	1.00	3.00	4.00	4.00	2.00
**DTR**	0.17	0.25	0.25	1.00	0.50	0.33	1.00	1.00	1.00	2.00
**Pop den**	0.25	0.50	0.50	0.33	0.20	0.25	1.00	1.00	2.00	4.00
**Prev**	0.25	0.50	0.50	1.00	1.00	0.25	1.00	0.50	1.00	2.00
**NDVI**	0.25	0.25	0.33	1.00	1.00	0.50	0.50	0.25	0.50	1.00

Tmean = average temperature, PP = precipitation, Al t = altitude, SLP = slope, DTWB = distance to water body, DTR = distance to road, NDVI = normalized difference index, LULC = land use and land cover, Mal prev = malaria prevalence, pop dens = population density.

**Table 4:** Consistency index interpretation.

Consistency Index (%)	Interpretation
0	Judgment is perfectly consistent
≤10	Consistent enough
≥10	Matrix needs improvement
≥90	judgments are random and are completely untrustworthy

## Appendix A

**Table A1:** Spectral characteristics of Landsat 8.

	Bands	Wavelength	Resolution
Landsat 8 Operational Land Imager (OLI) and Thermal Infrared Sensor (TIRS)	Band 1—Control aerosol	0.43–0.45	30
	Band 2—Blue	0.45–0.51	30
	Band 3—Green	0.53–0.59	30
	Band 4—Red	0.64–0.67	30
	Band 5—Near infrared	0.85–0.88	30
	Band 6—SWIR 1	1.57–1.65	30
	Band 7—SWIR 2	2.11–2.29	30
	Band 8—Panchromatic	0.50–0.68	30
	Band 9—Cirrus	1.36–1.38	15
	Band 10—Thermal infrared 1	10.60–11.19	100× (30)
	Band 10—Thermal infrared 2	11.50–12.51	100× (30)

Source: USGL.
